# *MUTYH* Associated Polyposis (MAP)

**DOI:** 10.2174/138920208785699562

**Published:** 2008-09

**Authors:** M.L.M Poulsen, M.L Bisgaard

**Affiliations:** Department of Cellular and Molecular Medicine, University of Copenhagen, 2200 Copenhagen N, Denmark

**Keywords:** Colorectal cancer, MUTYH associated polyposis, The MUTYH gene, base excision repair, (Attenuated) familial adenomatous polyposis, lynch syndrome.

## Abstract

*MUTYH* Associated Polyposis (MAP), a Polyposis predisposition caused by biallelic mutations in the Base Excision Repair (BER) gene *MUTYH*, confers a marked risk of colorectal cancer (CRC). The MAP phenotype is difficult to distinguish from other hereditary CRC syndromes. Especially from Familial Adenomatous Polyposis (FAP) and to a lesser extend Lynch Syndrome, which are caused by germline mutations in the *APC* and *Mismatch Repair (MMR)* genes, respectively.

Here we review research findings regarding *MUTYH* interactions, genotypic and phenotypic characteristics of MAP, as well as surveillance and treatment of the disease. The applied papers, published between 1/1 2002- 1/2 2008, were found through PubMed.

The exact role of *MUTYH* in CRC tumorgenesis is still uncertain, although MAP tumors show distinct molecular features, including somatic G:C>T:A transversions in the *APC* gene. Furthermore, cooperation between the BER and the MMR systems exists, as MUTYH interacts with MMR gene-products. Possibly, monoallelic defects in both pathways are of significance to CRC development.

Specific *MUTYH* variants are found to be characteristic in distinct ethnic populations, which could facilitate future genetic screening. Knowledge concerning functional consequences of many *MUTYH* germline mutations remains sparse. Most thoroughly investigated are the two most common *MUTYH* variants, *Y179C* and *G396D*, both generating dysfunctional gene products.

Phenotypic features of MAP include: development of 10-100 colorectal adenomas, debuting at 46-47 years, often CRC at time of clinical diagnosis, and in some, development of extracolonic manifestations.

## INTRODUCTION

Colorectal Cancer (CRC) is the second most prevalent cancer worldwide [1]. In 35% of CRC patients, statistically significant effects of hereditary factors have been found [2]. For some of these patients the genetic background is known; major CRC syndromes being: Lynch Syndrome, Familial Adenomatous Polyposis (FAP) and *MUTYH* Associated Polyposis (MAP), which will be focused on in this review. Fig. (**1**) shows a delimitation of the groups of patients, which are referred to in this paper.

Lynch Syndrome is characterized by the development of particularly CRC and endometrial cancer at a young age. Lynch Syndrome is an autosomal dominant disease often caused by germline mutations in one of the Mismatch Repair (MMR) genes [3, 5-7]. The clinical and genetic features of the syndrome have previously been thoroughly reviewed in [5-9].

Another autosomal dominant disease, Familial Adenomatous Polyposis (FAP), is caused by a germline mutation in the  *APC  *gene, and confers a near 100% risk of developing CRC. FAP has been shown to account for less than 0.1% of all CRC cases [10]. The characterization of the  *APC* gene and protein-product has been repeatedly reviewed, among others in [11] and [12]. Phenotypic characteristics of FAP include: early development of more than 100 and up to thousands of colorectal adenomas, as well as extracolonic manifestations such as gastric and duodenal adenomas, desmoid tumors and congenital hypertrophy of the retinal pigment epithelium (reviewed in [8, 13-16]). In FAP, genotype-phenotype correlations have been identified, specific *APC* gene mutations being associated with particular manifestations reviewed in [11] and [17]. Of particular clinical interest is the milder phenotypical FAP variant, Attenuated Familial Adenomatous Polyposis (AFAP), which is associated with  *APC* mutations in the extreme ends of, or in the alternatively spliced region of exon 9 [11, 18]. AFAP is distinguished from FAP by the development of less than 100 colorectal adenomas, fewer extracolonic manifestations and the later development of CRC [11, 13-16, 19]. One study showed that about 8% of registered FAP families present with an AFAP phenotype [19].

Prophylactic screening of Lynch Syndrome patients, FAP patients and their families is shown to reduce the development of CRC and CRC-associated mortality markedly [5, 9, 10, 13, 16, 20]. For Lynch Syndrome patients, the recommendation is colonoscopy from about 20-25 years of age, in intervals of 1-3 years [5, 7, 9, 20]. The benefit of screening for endometrial cancer and other cancers associated with Lynch Syndrome is still controversial, and recommendations should be adjusted according to the individual patient’s wishes, family history and possibly genotype [7, 9, 20].

Recommendations for FAP surveillance was recently reviewed in [13] and [16]. Sigmoidoscopy is advised to FAP patients commencing in the early teens, typically in intervals of 1-3 years, according to the clinical manifestations [13, 15, 16]. Furthermore, individual assessment is especially necessary in FAP families displaying a more severe phenotype [16]. Additionally, FAP patients should be offered endoscopy of the upper gastrointestinal tract from the age of about 25-30 years, in intervals of 1-5 years, according to the severity of duodenal Polyposis [16]. Moreover, prophylactic colectomy is often advisable for FAP patients. Especially in patients with an early first appearance of the disease, the surgical procedure recommended varies between the individual FAP patients [13, 15, 16]. For AFAP patients, coloscopy in intervals of 2 years is advised starting from 18-20 years of age, due to the later CRC development and the typically more distal location of adenomas in AFAP patients compared to FAP patients [16, 19].

In as many as 30% of patients with a FAP-like phenotype, no germline mutations in the *APC* gene can be found [15]. Similarly, one study of patients with an AFAP-like phenotype (3-100 adenomas), found that merely about 10% of these patients had inherited germline *APC* mutations [18]. However, another study (N=59) showed that almost 70% of patients with an AFAP-like phenotype (10-100 adenomas) had a germline *APC* mutation [19].

In 2002, the significance of mutations in the *MUTYH* gene regarding the development of the Polyposis predisposition syndrome *MUTYH* Associated Polyposis (MAP) was discovered [21]. Since then, many aspects of MAP have been investigated, and the important question of how, these new discoveries can be used in the genetic counseling and screening of individuals at risk of developing MAP, now stands to be answered.

Here we review the main genetic aspects of MAP, including analysis of functional consequences and the outlining of specific ethnic allelic frequencies of *MUTYH* variants. In addition, we review the clinical aspects of the syndrome and introduce a new nomenclature for *MUTYH* germline mutations, which is likely to replace the nomenclature, which is currently used.

We believe that this review provides a broad, up-to-date overview of existing findings regarding MAP. We hope to provide perspective of the significance of *MUTYH*, as well as of which issues regarding MAP call for future investigation.

## PATHOGENESIS OF MAP: THE *MUTYH* GENE AND BASE EXCISION REPAIR

### Base Excision Repair

The *MUTYH* gene product is part of the Base Excision Repair (BER) system, which serves as an important part of cells´ defense against oxidative damage to the DNA. The BER system and the functional role of MUTYH have previously been reviewed in detail by [22-31].

Oxidative DNA damage, caused by reactive oxygen species (ROS), which are produced during aerobic metabolism, exposure to certain chemicals or radiation, constantly threatens the integrity of cellular DNA. The oxidized base 7,8-dihydroxy-8-oxoguanine (8-oxo-G) is one of the most stable and mutagenic products of oxidative DNA damage. 8-oxo-G is often mistakenly paired with adenine (A), resulting in the appearance of Guanine:Cytosine > Thymidine:Adenine (G:C > T:A) transversions at the next round of DNA replication, as the detection of stable 8-oxo-G:A base-pairs is missed by the replicative DNA polymerases [22-31].

The human DNA-damage-specific glycosylases OGG1, MUTYH and MTH1, which are central enzymes in the BER pathway, function by specifically recognizing and facilitating the removal of 8-oxo-G [23-31]. The specific mechanism of recognition of the DNA-damage-specific glycosylases is characterized as “base-flipping”, involving the outwards rotation of nucleotides from the DNA helix. This allows the incorporated bases to be assessed by fitting into base-specific pockets of the glycosylases [22, 23, 26, 29-32]. The repair process at the damaged site is subsequently completed by the synthesis and incorporation of newly replicated DNA, involving several repair steps, which are facilitated by a sequence of DNA repair enzymes [23, 25, 26, 28-30]. The MUTYH glycosylase acts as the third level of BER, as it postreplicativly excises the misincorporated A opposite 8-oxo-G [23-25, 29-31]. For this reason, defective MUTYH function is associated with an increased frequency of G:C > T:A transversions [29]. Inactivation of MUTYH has accordingly been associated with various cancer forms [29], including lung cancer, gastric cancer, CRC [25, 33-36] and recently also endometrial cancer [37].

A number of studies of the human MUTYH glycosylase, both *in vitro* and *in vivo*, have demonstrated that the MUTYH glycosylase directly interacts with various proteins involved in other DNA repair pathways (reviewed by [29] and [31]).

Several studies have also screened the *OGG1* and *MHT1* genes in Polyposis patients without any significant findings of association with Polyposis or CRC phenotypes. [21, 38-41]. However, *OGG1* variants have recently been demonstrated to be significantly associated with a multiple adenoma phenotype [42] and the development of sporadic CRC [43], although the latter association was of borderline significance and should be further investigated [43]. This review focuses exclusively on the significance of *MUTYH* mutations in relation to MAP and CRC.

## THE *MUTYH* GENE

The MUTYH glycosylase is encoded by the *MUTYH* gene, located on the short arm of chromosome 1 (1p32.1- p34.3). The gene consists of 16 exons and encodes a protein of 535 amino acids, the MUTYH glycosylase. Characterization of the *MUTYH* gene and functional variants has been reviewed in [22-26, 29].

Exon 3 in *MUTYH* is alternatively spliced in various ways, generating different MUTYH transcripts [25, 29, 44]. In accordance with HGVS nomenclature rules, the longest *MUTYH* transcript existing (NM_012222.2 extended at the 5´of exon 3) will be used as coding DNA reference sequence, as outlined by Leiden Open Variation Database (LOVD) at http://www.LOVD.nl/MUTYH [44]. The use of the longest existing *MUTYH* transcript as coding DNA reference sequence is predicted to replace the previous commonly used *MUTYH* transcript (NM_001048171), although many authors still refer to this. Also, experts in this field (J. Sampson, F. Hes and S. Aretz) have recently discussed the use of *MUTYH* reference sequence. They agree that NM_012222.2 extended at 5´of exon 3 is the best option to use as reference sequence in the future [45].

Consequently, the amino acid numbering in this paper differs from the one used in many previous papers. An overview of the new and old terms regarding some of the most common *MUTYH* mutations mentioned in this paper can be found in Table **1**.

## DEVELOPMENT OF COLORECTAL ADENOMAS AND CRC TUMORGENESIS IN MAP

Somatic mutations in the  *APC* gene are important in CRC tumorgenesis due to the gatekeeper role of the  *APC* tumor suppressor gene, which is involved in many cellular processes. (Reviewed in [11] and [12].) The development of colorectal adenomas is likely to be initiated by an  *APC* gene left dysfunctional as a result of germline or somatic mutations [11].

Defects in the *MUTYH* gene were first shown to be associated with a Polyposis predisposition by Al Tassan  *et al*. in 2002 [21]. Defects in the BER genes were suspected, when 11 tumors from 3 related Polyposis patients showed somatic mutations in the  *APC  *gene, consistent with a defective BER system, while no germline  *APC* mutations were found [21].

The cause as to why, mutations in the *MUTYH* gene predispose to the development of colorectal adenomas in particular, has not yet been fully determined. However, the number of spontaneous somatic C:G > T:A transversions in the  *APC* gene is significantly greater in tumor cells with biallelic *MUTYH* germline mutations compared to tumor cells without *MUTYH* mutations [21, 38, 40, 46, 47].

The DNA sequence adjacent to the sites of G:C > T:A transversions in the 3’ end of the  *APC* gene appears to be of significance to the specificity of the MUTYH glycosylase. A significantly higher occurrence of GAA sites has repeatedly been demonstrated 3’ to the G:C > T:A transversions, thus creating stop codons, and resultantly a truncated APC  protein. These results are seen even though the G:C > T:A transversions theoretically could occur at any other G:C site in the  *APC* gene [21, 38, 48]. Also,  *in vitro * experiments with  *E. coli  *MutY have revealed a pronounced sequence preference for MutY to GAA [49]. However, the significance of these findings and the question as to why GAA sites seem to be prone to G:C > T:A transversions, remain unclear and call for further investigations.

Both the high number of GAA sites in the  *APC* gene compared to other key tumorgenesis genes frequently involved in other cancers [23], as well as the considerable exposure to ROS in the gastrointestinal tract, could be part of the explanation of why germline *MUTYH* mutations, and subsequently somatic APC mutations, are associated with development of particularly CRC [23].

Characteristic molecular profiles of colorectal tumors, both adenomas and carcinomas, taken from MAP patients have been found [46, 50]. Distinct features of MAP tumors include: C:G > T:A transversions in the  *APC  *gene and the proto-oncogene  *K-Ras * [46, 50]. Comparable with somatic  *APC* mutations, statistically significant numbers of somatic mutations in the *K-Ras* gene have been found in MAP tumors, compared to tumors without *MUTYH* mutations [47, 50]. The described *K-Ras* mutations have all been identical G > C transversions in codon 12, *G12C* [46, 47].

Molecular features in MAP tumors are characteristic to these, compared to carcinomas from Sporadic CRC, FAP or Lynch Syndrome tumors [46], which can potentially be used in classification of CRCs [50]. These molecular characteristics of MAP carcinomas include: low MSI (Microsatellite instability), low frequencies of  *APC, β-cantenin* mutations and LOH (Loss of Heterozygosity) of 18q, harboring the *SMAD4* gene, and the karyotype of tumor cells typically being near-diploid [46, 50].

The issue of a possible significance of the *MUTYH* gene in Sporadic CRC has been only sparsely addressed, and with contradictory outcomes. Halford *et al*. found no indications of *MUTYH* involvement in Sporadic CRC [39]. In contrast, somatic mutations in the *MUTYH* gene have recently been demonstrated in Sporadic CRC, indicating a role of the *MUTYH* gene in Sporadic CRC tumorgenesis [51].

## INTERACTION BETWEEN THE *MUTYH* GENE AND THE MISMATCH REPAIR GENES

The Mismatch Repair (MMR) system functions postreplicativly correcting DNA errors, which occur during DNA replication. The normal MMR function and characterization of defects in the MMR system have previously been reviewed in [7, 29] and [52]. Key proteins in the human MMR system include MutL homologs (MTH1 and Pms2) and MutS homologs (MSH2, MSH3, MSH6), the latter group forming two heterodimeric complexes [7, 29, 52].

Presumably cooperation between the BER and the MMR systems exists, since *in vitro* experiments have shown that MUTYH physically interacts with the MSH2/MSH6 heterodimeric complex *via* a hMSH6-binding domain [53, 54]. These two studies have further demonstrated, that the MSH2/MSH6 complex stimulates the activity of the MUTYH glycosylase by enhancing the affinity of MUTYH for 8-oxo-G:A mismatched base pairs in the DNA [53, 54].

Several *MUTYH* germline mutations are shown to influence the interaction between MUTYH and MSH6, followed by a massive decrease in activity of the MUTYH protein [54, 55]. Mutations in one or more of the genes involved in the two systems possibly affect the repair of DNA damage caused by 8-oxo-G. The importance of this in regard to CRC tumorgenesis is still uncertain, although there have been indications, that interaction between a defect *MUTYH* gene and a defect MMR gene is of significance in regard to CRC risk. In a study by Niessen *et al*. (N=210), a significantly higher frequency of carriers of monoallelic *MUTYH* mutations was found among CRC patients who also had a specific MMR mutation (5/36=14%), in comparison to groups of CRC patients with other MMR mutations (1/40= 2.5%) or without MMR mutations (1/134 =0.7%) [56]. In this study a particularly strong association between monoallelic *MUTYH* germline mutations and en missense variant of the *MSH6* gene was found (4/20 = 20%) [56], consistent with the before mentioned interaction between the two corresponding proteins.

In contrast, studies of CRC patients have indicated that the BER and MMR pathways may be mutually exclusive, although none have found significant results [48, 57]. Furthermore, Van Puijenbroek *et al*. found a remarkably mild Polyposis phenotype in a patient both compound heterozygote for *MUTYH* mutations, as well as being a carrier of a *MSH6* germline mutation, supporting this notion [58]. However, these studies do not provide substantial data for any conclusions, for which reason further investigations need to be carried out.

## ALLELIC FREQUENCIES OF MUTYH GERMLINE MUTATIONS IN DIFFERING POPULATIONS

An overview of the most commonly identified germline mutations in the *MUTYH* gene to date can be found at the LOVD at http://www.LOVD.nl/MUTYH.

Specific mutations in the *MUTYH* gene are found in different populations, see Fig. (**2**). In European populations the two missense mutations *Y179C* and *G396D* are most frequently seen, and have solely been found in Caucasians. The allelic frequencies of *Y179C* and *G396D* found among MAP patients are much higher compared to those found in background populations, see Fig. (**3**).

In Asian populations, *Y179C* and *G396D* do not seem to be of significance with regard to the development of Polyposis, since neither has been found in Asian Polyposis patients or in the corresponding background populations [42, 43, 55, 59-61]. In studies of Korean and Japanese Polyposis patients (N=97), 7.2% were biallelic carriers of other germline mutations in the *MUTYH* gene [55, 61]. Other studies of Korean and Singaporean Polyposis patients (N= 63) failed to find any *MUTYH* mutations after screening of coding regions in the entire gene, although these results may be biased due to small sample size [43, 59, 60]. Characteristic mutations found in Japanese Polyposis patients include the missense mutation *R245C* and the splice-site mutation *IVS10-2A>G*, neither of which were found in the corresponding background population [55].

To date, five unrelated Indian MAP patients have been identified, all were homozygote for the missense mutation  *E480X * [38, 62, 63]. However, in a case-control study of Indian Polyposis patients (cases: N=120 and controls: N=100), merely one case and one control were found to be heterozygote for *E480X*, while no other *MUTYH* mutations were found [64]. These results suggest that *MUTYH* mutations are unlikely to be of significant importance to development of Polyposis among Indian individuals. Other *MUTYH* variants found in noteworthy allelic frequencies in characteristic populations are: *c.1145delC* (found in Italian MAP patients in allelic frequencies of 0.07-0.11 [65, 66]), *A473D* (found in Finnish Polyposis and CRC patients, in the latter group with an allelic frequency of 0.01 [67]) and *E383fsX451* (found in Portuguese MAP patients with allelic frequencies of 0.15-0.19 [68, 69]).

In addition, other *MUTYH* polymorphisms without any apparent pathogenic importance have been found. The most frequent of these are *V22M*, *Q388H* and *S515F*, the allelic frequencies of which in healthy control groups are found to be equivalent to those found among MAP patients [21, 39-41, 48, 56, 67, 69-78].

## FUNCTIONAL CONSEQUENCES OF SPECIFIC GERMLINE *MUTYH* MUTATIONS

The two most common *MUTYH* mutations, the *Y179C* and *G396D*, are situated in the catalytic and C terminal domains of MUTYH, respectively. Both of these MUTYH residues have important roles in the recognition of 8-oxo-G in A:8-oxo-G mispairs [31, 32, 49, 79, 80]. Accordingly, functional studies of murin variants corresponding to *Y179C* and *G396D* have indicated compromised substrate recognition as a consequence of these mutations [81].

The *Y179C* mutation is located in the N-terminal end of the MUTYH, and is part of the pseudo-HhH (helix-hairpin-helix) motif in the catalytic region of the MUTYH. This region is thought to promote the base-flipping mechanism in substrate recognition, participate in maintaining stability during this process, as well as being involved in DNA binding [30, 49, 79, 82] (thoroughly reviewed in [31]). Studies of biallelic *Y179C* mutations in human cell lines illustrate, that defective MUTYH function results from both significantly reduced levels of MUTYH protein (Protein levels of 5-10% compared to wild-type MUTYH levels) as well as from reduced binding and cleavage ability towards the mispaired substrates [83].

*G396D* is located in the C-terminal domain of the MUTYH, which is thought to be responsible for 8-oxo-G recognition and binding, as well as mediating the base-flipping mechanism [31, 32, 49, 79, 80]. Human cell lines with biallelic *G396D* mutations show defective MUTYH function as a result of the production of a dysfunctional protein. The protein shows both reduced binding activity of mispaired substrates (about 50% of the wild-type MUTYH activity), as well as lower rates of repair, compared to the wild type MUTYH [83]. However, the MUTYH protein levels in these cell lines were found to be equivalent to the levels of wild type protein, indicating that protein instability is not a consequence of *G396D* mutations [83].

These results are consistent with previous studies of the corresponding  *E. coli* *MutY* variants, which have been shown to severely compromise the activity of the glycosylase [21, 49]. In one study, the *Y179C* and  *G396D* variants showed a 98% and a 86% reduction in adenine removal from a G:A substrate, compared to the wild-type protein, respectively [21]. Likewise, both  *MutY* variants exhibit significantly reduced rates of adenine removal compared to wild-type MutY. The variants corresponding to *G396D* and *Y179C,* showing a 6-fold and 80-fold slower rate, respectively [49]. Also, considerably reduced binding affinities for G and 8-oxo-G substrates were observed in the cases of both variants [49].

Functional studies of additional *MUTYH* mutations have also been conducted, although none as comprehensive as for *Y179C* or *G396D*, for which reason merely a short overview will be given in the present review:  *R182C*, *R182H*, *R185Q* and *G189E*, all are located in the pseudo-HhH motif of the MUTYH catalytic domain, are considered to induce functional MUTYH changes comparable to those observed in cell lines with *Y179C* variants [57, 69, 74, 75]. The missense variants *P405L* and *A473D*, both located in the C-terminal domain, are both supposed to have functional significance [67, 74].

Functional analyses of two *MUTYH* mutations, which lie close to or within the MSH6-binding domain of the *MUTYH* gene, have been preformed: The variants *R241W* and *V246F* have preserved their ability to physically interact with MSH6, but both show reduced MUTYH function [54]. *R245C,* also located near the MSH6 binding domain, is likewise assumed to compromise the interaction between the MUTYH and MSH6 [55].

## THE SIGNIFICANCE OF THE *MUTYH* GENE IN RELATION TO THE DEVELOPMENT OF POLYPOSIS

Results from several studies indicate, that mutations in the *MUTYH* gene, more often than previously assumed, are the disease causing factor in Polyposis patients. Sieber and colleagues found, that an AFAP-like phenotype is more often caused by germline mutations in the *MUTYH* gene than in the *APC* gene [40]. Another study of patients with AFAP phenotypes, found *APC* germline mutations and *MUTYH* biallelic mutations in equal numbers of families [19]. Consistent with these results, a study of CRC patients by Enholm *et al*. has suggested, that the contributions of germline mutations in the *APC* gene and the *MUTYH* gene are fairly equal [84].

In studies of Polyposis patients (N=995), it has been found that 5-22% of the patients with 3-100 adenomas and 7.5-17% of those with over 100 adenomas had biallelic mutations in the *MUTYH* gene [19, 40, 85]. Furthermore, several studies have found that none of the patients, who had biallelic germline *MUTYH* mutations (N=63), presented with a phenotype consistent with severe classical FAP, the criteria being: more than 1000 polyps or early-onset CRC (before the age of 50 years), and the development of more than 100 polyps before the age of 35 years, respectively [40, 85, 86]. These results indicate that a higher proportion of AFAP-like phenotypes are caused by *MUTYH* mutations compared to FAP-like phenotypes.

## CLINICAL FEATURES OF MAP

Table **2** provides an overview of phenotypic characteristics of MAP, using information gathered from different studies of MAP patients.

## MODE OF INHERITANCE

MAP is an autosomal recessive disease, caused by biallelic mutations in the *MUTYH* gene. The majority of family histories of MAP patients were found to be consistent with a recessive inheritance, typically with affected siblings, but unaffected parents [19, 21, 38-40, 62, 65, 66, 68, 69, 74, 75, 85-87]. Furthermore, Russel *et al*. found no *MUTYH* germline mutations among Polyposis patients, who were negative for *APC* germline mutations, and had a family anamnesis consistent with a dominant mode of inheritance [75]. In contrast, other studies have found family histories appearing to follow a dominant mode of inheritance among MAP patients in about 15-30% of the studied cases [68, 69, 74, 78, 85]. However, it is possible, that a recessive trait as MAP, due to a relatively high frequency of heterozygote mutation carriers in some populations, can mimic dominant inheritance, displaying a pseudo-dominant mode of inheritance, especially in cases of parental consanguinity. On the other hand, some have proposed a co-dominant model for mode of inheritance, suggesting an increased CRC risk for monoallelic *MUTYH* germline mutation carriers compared to non-carriers, as will be discussed further [48, 57, 70, 71, 84, 88-92].

In studies of healthy controls, no unaffected individuals, who were homozygote for germline *MUTYH* mutations, have been found [57, 70, 71, 84, 90, 91] and Table **3**. This indicates that biallelic germline *MUTYH* mutations are highly penetrant. Accordingly, in a case-control study of CRC patients (N=2,239), all homozygote *MUTYH* carriers (N=12) developed CRC before the age of 60 years [92].

## AGE AT TIME OF CLINICAL DIAGNOSIS OF MAP

The average age at time of clinical diagnosis among MAP patients is typically around 47 years (range: 13-72 years, N=106) [19, 57, 62, 76, 77, 85-87] and Table **2**.

Characteristically, the age at time of clinical diagnosis is higher among patients with biallelic *MUTYH* germline mutations compared to Polyposis patients without *MUTYH* mutations. This applies both to studied Polyposis patients with <100 adenomas [57, 65, 74] as well as to Polyposis patients with >100 adenomas, who are also negative for *APC* germline mutations [40, 93]. In one of these studies (N= 58) this result was statistically significantly [74].

Because of the recessive mode of inheritance, the identification of MAP patients is complicated, as MAP patients often seem to be sporadic cases with no family history of the disease at clinical presentation. Consequently, most MAP patients are diagnosed due to symptoms and not as a result of prophylactic screening, unlike many FAP and AFAP patients [85]. In accordance to this, many MAP patients are also typically discovered at a later time in the course of their disease than other groups of Polyposis patients. For example, this can be illustrated by the higher proportion of MAP patients, who have already developed CRC at the time of their clinical diagnosis as compared to FAP patients (see later).

## DEVELOPMENT OF ADENOMAS

In several studies of Polyposis patients, the phenotypes in regard to the number of adenomas are generally seen to be more severe in MAP patients compared to the phenotypes of AFAP patients, but milder compared to those of classic FAP patients [40, 61, 68, 74, 85].

Characteristically, MAP patients develop between 10-100 adenomas (Table **2**), which is a smaller number than seen in the classic FAP phenotype. Several studies of Polyposis patients have all found the highest incidence of biallelic *MUTYH* mutations in groups of patients with between 15-100 adenomas [40, 57, 61, 69, 85]. In the applied studies, the incidences of biallelic *MUTYH* mutations in the groups of patients having 15-100 adenomas, were found to be between 16-47% (N=835). However, none of the results were statistically significant.

In a recent study of AFAP patients (N=140), comparing the clinical features of patients with *APC* (N=93) and biallelic *MUTYH* germline mutations (N=26), no significant differences between the two groups were found [19]. However, this result might be biased due to the smaller sample size of the MAP patients compared to the patients with germline *APC* mutations. When compared to Polyposis patients without mutations in neither the *MUTYH* nor the *APC* gene, MAP patients seemed to develop the lowest number of adenomas, although no statistically significant results have been found [40, 77].

The morphology of the adenomas appears to be similar regardless of whether their occurrence is caused by germline mutations in the *MUTYH* or in the *APC* gene [40, 65]. Likewise, microadenomas have been found in patients with both genotypes [40, 46, 62].

## DEVELOPMENT OF CRC

In several studies of CRC patients (N=3,320), biallelic germline *MUTYH* mutations were found in 0.4-1.9% of all cases (Table **3**). Based on these results, the contribution of biallelic *MUTYH* mutations to CRC seems to correspond to, or even be greater than that of FAP, FAP accounting for less than 0.1% of all CRC cases, as found by Bülow [10].

In two other studies of CRC patients (N= 2,268), a significant association between biallelic germline mutations in the *MUTYH* gene and the development of CRC, has been determined [75, 92]. In one of these studies (N=2,239), MAP patients were found to have a 93-fold increased risk of developing CRC compared to a group of unaffected controls from the general population [92].

Among MAP patients, the average age of CRC onset is found to be 47 years (range: 29-72 years) [19, 62, 68, 75, 85, 87]. The frequency of patients with a synchronous CRC at time of diagnosis is greater among MAP patients compared to among FAP patients [69, 74, 93]. These results comply with the fact that FAP patients generally are diagnosed earlier than MAP patients, facilitated by the dominant mode of inheritance of FAP and the use of Polyposis Registers in many countries. Therefore, the prophylactic treatment of FAP prevents the development of CRC in a higher number of patients. As both probands and call-ups were included in the applied studies, and as none of the results were statistically significant, more specific studies examining only probands are needed.

There have been many inconsistent results regarding the typical location of carcinoma in MAP patients. Overrepresentation of both right and left sided CRC has been demonstrated [19, 39, 46, 71, 74, 75, 87, 88]. Presumably, the location of CRC among MAP patients should not be considered important, as the prognosis seems to be independent of the CRC location [74].

## ASSOCIATED CANCERS AND EXTRACOLONIC MANIFESTATIONS

The manifestation of other primary cancers than CRC or other extracolonic manifestations are less frequent among MAP patients compared to among FAP patients [74]. Several studies have failed to report other cancers than CRC or any extracolonic features among the examined MAP patients [38, 40, 57, 61, 65, 66, 69, 71, 93]. The methods of clinical examination were not specified in the applied studies. For this reason, bias could be suspected, as patient information is often gathered from several different databases without assurance that all patients were systematically examined.

Conversely, several studies have described the occurrence of extracolonic features in MAP patients, mostly upper gastrointestinal lesions [37, 40, 62, 63, 74, 75, 78, 84-87, 94-96], see Table **4**.

However, these results should be regarded with reservations, as the numbers of examined MAP patients in the majority of the studies were very small. In addition, bias could result from a difference in the methods of investigation used in the individual studies, as these are only sparsely describe in most of the studies. Also, some of the reported extracolonic manifestations were reported in very low frequencies among the examined MAP patients. They are therefore more likely to be present by chance, rather than being associated with biallelic *MUTYH* mutations.

The described findings suggest that extracolonic manifestations are generally not a part of the characteristic MAP phenotype, but can occur. However, further studies with more systematic and thorough investigation of MAP patients are needed to address this issue.

## HETEROZYGOTE AND CRC RISK

At present, no conclusive evidence has been found, that monoallelic carriers of *MUTYH* germline mutations have an increased CRC risk compared to the general population. However, as seen in Table **3**, percentages of carriers of monoallelic germline *MUTYH* mutations are generally larger among CRC patients, than the same percentages among the corresponding background populations. Furthermore, several studies have shown a tendency for a slightly elevated CRC risk [40, 41, 57, 62, 66, 70, 71, 85, 88, 90, 93, 97], especially in those over 55 years of age [41, 89, 92, 98]. However, reservations towards these studies should be taken, as results from the mentioned studies have failed to be convincing, with merely one study achieving a slight statistical significance [97]. Also, some of the mentioned studies have been criticized for the statistical methods used [98, 99], and meta-analyses of the different studies have found inconsistent results [89, 98].

A suggested explanatory model for a possible association between monoallelic *MUTYH* mutations and a co-dominant mode of inheritance of CRC, is LOH of chromosome 1p, where the *MUTYH* gene in located, possibly representing an early event in CRC tumorgenesis [48, 70]. According to this model, loss of the wild-type *MUTYH* gene on 1p in monoallelic *MUTYH* mutation carriers is likely to contribute to an increased CRC risk, as 1p LOH has been found in tumors from monoallelic *MUTYH* mutation carriers [48, 77]. In contrast, other studies that have investigated 1p LOH in tumors from monoallelic *MUTYH* mutation carriers have failed to find results of sufficient significance to support this theory [40, 84].

On the other hand, Peterlongo *et al*. combined results from 9 case-control studies of CRC patients (Cases: N= 2,707 and controls: N= 2,321), and were not able to demonstrate a significant association between monoallelic carriers of *MUTYH* germline mutations and the development of CRC [91]. Consequently, as it –in the worst of cases- can only be a matter of a minimally increased CRC risk compared to the risk of the general population, it seems unlikely, that the tendency for an increased CRC risk in heterozygote individuals is powerful enough to be of diagnostic or prophylactic importance.

The exact role of monoallelic *MUTYH* germline mutations in CRC tumorgenesis is still uncertain, but as mentioned earlier, interactions with other genes, for example a MMR gene, are possibly of significance.

In addition, several large studies of Polyposis patients have found frequencies of monoallelic *MUTYH* mutation carriers that correspond fairly well to those of biallelic carriers, see Table **5**.

This indicates that monoallelic *MUTYH* germline mutations may be associated with a Polyposis phenotype. However, as seen in Table **5**, the reported frequencies of mono-and biallelic carriers vary considerably among studies. This is likely to be a result of differing inclusion criteria, as these are not thoroughly described in all of the applied studies. The significance of monoallelic *MUTYH* mutations needs to be assessed further in comparable studies on the subject.

## GENETIC COUNSELING AND PROPHYLAXIS

Frequently, MAP patients have already developed CRC at the time of clinical diagnosis, before prophylactic treatment can be initiated. Genetic testing and counseling of individuals at risk of developing MAP, is essential for the future prospect of MAP patients, so that prophylactic screening can be initiated. In this context it is important that knowledge about the disease and mode of inheritance is continuously searched for and utilized for the organization of guidelines, which we believe will assure the best treatment for these patients.

## DETERMINATION OF THE GENOTYPE

Based on the recessive mode of inheritance most commonly seen in MAP families, siblings to an affected individual have a 25% a priori risk of disease. Consequently, determination of the genotype is especially important in these individuals [62, 75, 92]. In some MAP families, the mode of inheritance is pseudo-dominant, i.e. appears to be dominant although in reality recessive [19, 68, 74, 87].

In practice, it is important to search for germline mutations in both the *APC* and the *MUTYH* gene. This applies to both individuals having a familiar disposition for multiple adenomas and/or CRC as well as in apparently sporadic CRC cases, if the clinical presentation gives hints of a Polyposis syndrome. In cases with a positive family anamnesis, the most probable mode of inheritance can guide the assessment of which gene to start with, i.e. the *APC* or the *MUTYH* gene when a dominant mode or recessive mode is seen, respectively [19].

Furthermore, the characteristic mutations in specific populations makes it possible to target the MAP screening in accordance with ethnic background, thereby making the screening more efficient, when the background of the patient is known [57, 64].

As *Y179C* and *G396D* at present are the most frequently found mutations in Caucasian MAP patients, an obvious possibility would be to screen specifically for these in Caucasian individuals [65]. A disadvantage of such a selective screening is, that MAP patients, who are compound heterozygote for just one of these variants, or homozygote for other *MUTYH* variants, would be missed [57, 77, 86, 100]. Eliason *et al*. demonstrated an increased clinical sensitivity for the detection of *MUYTH* mutations in their study, when all exons and intron-exon boundaries of the *MUTYH* gene were screened, compared to the sole testing for Y179C and G396D [100]. Accordingly, all coding regions of the *MUTYH* gene should be screened in individuals found to be heterozygote for *Y179C* or *G396D* to establish their true genetic status [85, 86, 100]. Recently, Piccioli *et al*. have designed specific assays for detecting the 6 most frequently found *MUTYH* mutations using a multiplex T-ARMS-PCR method [101]. This method has been shown to be both accurate and inexpensive, and can furthermore be adapted according to the specific frequencies of *MUTYH* mutations in different population groups [101]. In patients presenting with an atypical MAP phenotype, i.e. <10 colorectal adenomas or familial mismatch repair proficient CRCs, van Puijenbroek *et al*. have proposed a prescreening method also considered to be cost-effective [102]. This method consists of the screening of tumors for *KRAS2 c.34G > T*, a somatic mutation in the *KRAS2* gene shown to be more common in MAP patients compared to sporadic CRC cases. This should be followed by screening for population specific *MUTYH* mutations in cases positive for the aforementioned *KRAS2* mutation [102].

The frequencies of *Y179C* and *G396D* in the general population are low compared to the occurrence among MAP patients (Fig. (**2**) and (**3**)), and for this reason, there is at present no indications for MAP screening of the general population [75, 84, 85]. However, genetic testing of spouses of *MUTYH* mutation carriers to asses the genotype and corresponding disease risk of offspring, has been recommended [77].

## PROPHYLAXIS AND TREATMENT

The prophylactic surveillance of MAP patients is recently reviewed by Vasen *et al*. in [16]. Here a surveillance protocol in accordance with the recommendations for AFAP patients is suggested [16]. However, some recommend beginning at the age of 20-25 years, which is later compared to AFAP recommendations [19]. The surveillance of MAP patients should consist of colonoscopy in two-yearly intervals as opposed to sigmoidoscopy in FAP patients, due to the often more attenuated phenotype and distal polyp location of MAP compared to FAP [16, 19]. Furthermore, upper gastrointestinal endoscopy starting from the age of 25-30 years is advised in MAP patients [16], even though the question of upper gastrointestinal endoscopy ought to be further investigated in studies, more specifically researching extracolonic manifestations in MAP patients.

Naturally, the outlined recommendations should be adjusted according to the number, size and degree of dysplasia of the adenomas of the individual patient [57].

Since MAP patients typically develop less adenomas than FAP patients, the prophylactic treatment of MAP patients should as a starting point be aimed at colonoscopy with polypectomy. It could however be appropriate to apply colectomy in MAP patients developing a particularly large number or advanced adenomas [16, 19, 68].

## GENETIC COUNSELING OF HETEROZYGOTE CARRIERS OF *MUTYH* GERMLINE MUTATIONS

In our judgment, there is no indication for prophylactic surveillance in heterozygote carriers of *MUTYH* germline mutations at the present time. However, we believe that relatives at risk of developing MAP should be searched for on the basis of family anamnesis, if *MUTYH* germline mutations are discovered, in order for appropriate measures to be made on this basis.

## CONCLUSION AND FUTURE PERSPECTIVES

The full extent of the significance of *MUTYH* in the development of CRC is yet to be resolved. Biallelic germline mutations in the *MUTYH* gene are found to be associated with a markedly increased risk of developing Polyposis and CRC. The interactions between *MUTYH* and the MMR system could play a role in the CRC tumorgenesis in MAP patients.

At times, it can be difficult to distinguish between the phenotypes of FAP, AFAP, Lynch syndrome and MAP. Aspects regarding phenotypic differences between MAP patients and other Polyposis patients form the base of the recommendations for counseling and prophylactic treatment of MAP patients, which is stated here. Germline mutations found in the *MUTYH* gene have shown a great ethnic variability, and further knowledge about this could be used to target the genetic screening of Polyposis patients towards specific population groups. Genetic screening for germline mutations in the *MUTYH* gene as well as in the *APC* gene should be performed on equal terms, perhaps guided by the most probable mode of inheritance. Prophylactic surveillance of MAP patients could be colonoscopy with polypectomy in mind from 20-25 years of age.

In the future, more MAP patients could be identified before developing CRC by establishing MAP registers and finding call-up patients based on family anamnesis, as it is currently done for FAP patients in many countries. In this way the future prospects of MAP patients could be improved markedly.

## SEARCH CRITERIA

The applied papers were all found in the PubMed database using the following terms: MYH Associated Polyposis /MUTYH Associated Polyposis /MutYH Associated Polyposis. The search was only for material published in English and was made without time-limitation. All papers published between 01/01 2002 and 01/02 2008. We also searched reference lists of relevant papers.

## Figures and Tables

**Fig. (1):**
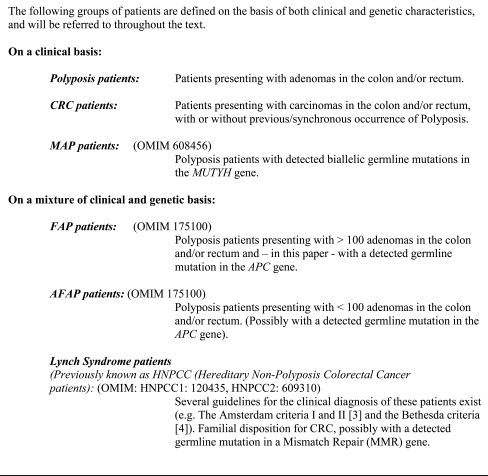
Delimitation of the groups of patients.

**Fig. (2) Mean Allelic Frequencies among Carriers of MUTYH Germline Mutations.**
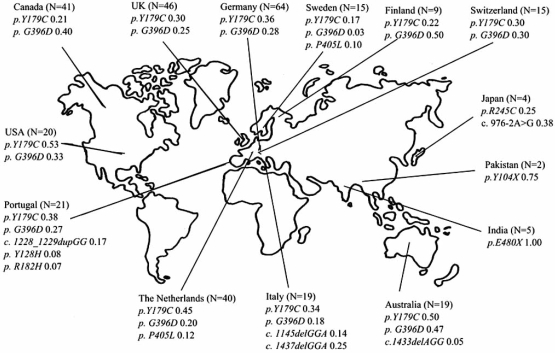
N= Number of biallelic and monoallelic *MUTYH* germline mutation carriers. Included are mutations which are believed to be of pathogenic significance, and which are found with an allelic frequency of > 0,03 in mutation carriers from the respective countries. The figure is based on data colleced from the following studies: Al-Tassan *et al.*, 2002; Jones *et al.*, 2002; Sieber *et al.*, 2003; Sampson *et al.*, 2003; Halford *et al.*, 2004; Fleischmann *et al.*, 2004; Gismondi *et al.*, 2004; Isidro *et al.*, 2004; Venesio *et al.*, 2004; Wang *et al.*, 2004; Aceto *et al.*, 2005; Kairupan *et al.*, 2005; Miyaki *et al.*, 2005; Leite *et al*., 2005; Aretz *et al.*, 2006; Kanter-Smoler *et al.*, 2006; Niessen *et al.*, 2006; Russel *et al.*, 2006, Ajith Kumar *et al.*, 2007.

**Fig. (3) Mean Allelic Frequencies of p. Y179C and p. G396D in Background Populations.**
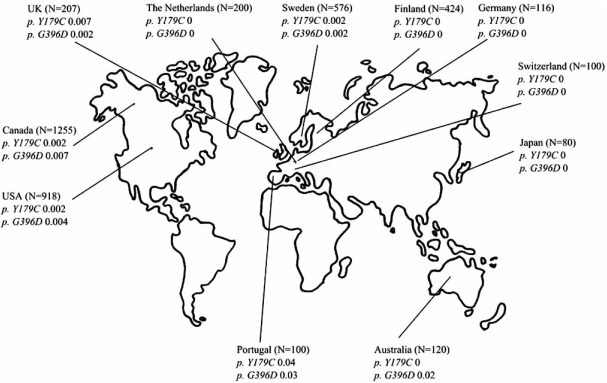
N= Number of tested individuals. The individuals tested belong to control groups without Polyposis. The figure is based on data colleced from the following studies : Al-Tassan *et al.*, 2002; Enholm *et al.*, 2003; Sieber *et al.*, 2003; Croitoru *et al.*, 2004; Isidro *et al.*, 2004; Leite *et al.*, Miyaki *et al.*, 2005; Peterlongo *et al.*, 2005; Zhou *et al.*, 2005; Aretz *et al.*, 2006; Kairupan *et al.*, 2005; Kanter-Smoler *et al.*, 2006; Niessen *et al.*, 2006; Russel *et al.*, 2006.

**Table 1.** Overview of Nomenclature for *MUTYH* Germline Mutations

Nomenclature from Reference Sequence NM_ 001048171	Nomenclature from Reference Sequence NM_012222.2 (Extended at the 5’ end of exon 3)*
*p.V22M *	*p.V22M*
*p.Y90X*	*p.Y104X*
*p.Y114H*	*p.Y128H*
*p. Y165C*	*p.Y179C*
*p.R168H *	*p.R182H*
*p.R168C*	*p.R182C*
*p.R171Q*	*p.R185Q*
*p.G175E*	*p.G189E*
*p.R227W*	*p.R241W*
*p.R231C *	*p.R245C*
*p.V232F*	*p.V246F*
*p.Q324H*	*p.Q338H*
*p.E369fsX437*	*p.E383fsX451*
*p. G382D*	*p. G396D*
*p.P391L*	*p.P405L*
*p.A459D*	*p.A473D*
*p.E466X*	*p.E480X*
*p.S501F*	*p.S515F*
*c.934-2A>G*	*c.976-2A>G*
*c.1391delAGG *	*c.1433delAGG*
*c.1103delGGA*	*c.1145delGGA*
*c.1395delGGA *	*c.1437delGGA*
*c.1103delC*	*c.1145delC*
*c.1186_1187insGG *	*c.1228_1229dupGG*
*c.IVS10-2A>G *	*c.IVS10-2A>G*

* The new nomenclature for *MUTYH* mutations is based on referral to the longest existing *MUTYH* transcript and were found at http://www.LOVD.nl/MUTYH

**Table 2.** Clinical Features of Identified MAP Patients

	Sieber *et al*. 2003 [40]	Sampson *et al*. 2003 [62]	Isidro *et al*. 2004 [69]	Wang *et al*. 2004 [57]	Nielsen *et al*. 2005 [87]	Russell *et al*. 2006 [75]
Number of MAP patients (Carriers of biallelic *MUTYH* mutations)	N=14	N=25	N=21	N=16	N=40	N=7
Mean age at the time of clinical diagnosis	For Patients with 10-100 adenomas: 56 years Range: 45-59 years For Patients with 100-1000 adenomas: 48 years Range: 30-70	46 years Range: 13-65 years	50 years Range: 36-68 years	47 years Range: 37-63 years	45 years Range: 21-67 years	48 years Range: 33-60 years
Number of colorectal adenomas	43%: Median: 55 Range: 18-100 57%: 100-1000	44%: 10-100 36%: >100 20%: Unspecified	71%:10-100 19%:100-1000 5%: >1000	19%: 20-49 19%: 50-99 25%: 100-500 35%: Unspecified	29%: 10-99 42%:“Multiple” 29% 100-1000	<100 (Only patients with 5-99 adenomas were tested)

In this table only studies in which the numbers of tested MAP patients are > 5 are included. The tested individuals are all APC germline mutation negative. The individuals tested are either probands or call-up patients with colorectal adenomas.

**Table 3.** The Frequencies of Carriers of MUTYH Germline Mutations Y179C and G396D Among CRC Patients and Back-Ground Populations

	Enholm *et al*. 2003 [84]	Croitoru *et al*. 2004 [70]	Fleischmann *et al*. 2004 [71]	Wang *et al*. 2004 [57]	Peterlongo *et al*. 2005 [91]	Webb *et al*. 2006 [98]	Küry *et al*. 2007 [41]
Population group	Finland	Canada	UK	USA	USA	UK	France
**Carriers of *MUTYH* germline mutations among CRC patients**							
Number of tested individuals with CRC	N =1042	N=1238	N =358	N=444	N=238	N=2561	N=1024
Monoallelic carriers	0.5 %	2.3 %	2.2 %	2.3 %	1.7 %	2.1 %	2.3 %
Biallelic carriers	0.4 %	1.9 %	0.6 %	0.5 %	0.8 %	0.2%	0.1%
Total percentage of carriers of *MUTYH* germline mutations	0.9%	4.2%	2.8%	2.8%	2.5%	2.2%	2.4%
**Carriers of *MUTYH* germline mutations in back-ground populations**							
Number of individuals tested	N= 424	N= 1255	N=207*		N=918	N=2695	N=1121
Monoallelic carriers	0	1.7%	1.9%		0.8%	2.11%	1.8%
Biallelic carriers	0	0	0		0	0	0

In this table only studies in which the numbers of tested individuals are >  50, are included.

* Based on data from studies by Al-Tassan *et al*., 2002 and Sieber *et al*., 2003.

**Table 4.** Extracolonic Manifestations Reported in MAP Patients

Extracolonic Manifestation	Number of MAP Patients (with Biallelic *MUTYH* Mutations) Examined	Overall Percentage of MAP Patients with Extracolonic Manifestations	Reported by
Duodenal lesions (Adenomas and/or cancer)	N= 154	13%	[40, 62, 74, 75, 77, 85, 86, 87, 88, 94]
Gastric lesions (Fundic gland polyps or stomach cancer)	N= 133	8%	[62, 85, 86, 87, 88]
CHRPE (Congenital hypertrophy of the retinal pigment epithelium)	N= 22	18%	[40, 93]
Osteomas	N=14	14%	[93]
Desmiod cysts	N=14	7%	[93]
Oesophageal cancer	N=16	6%	[87]
Thyroid carcinoma	N= 57	4%	[85, 95]
Breast cancer	N=22 (female MAP patients)*1	18%	[87]
Dental cysts	N=14	7%	[93]
Tooth agenesis	N=7	14%	[78]
Lipoma	N=56	4%	[85]
Multiple sebaceous adenomas	N=2 (case reports)	100%	[63, 95]
Sebacous carcinoma	N=1*2 (case reports)	100%	[37]
Pilomatricomas	N=2 (case reports)	100%	[96]
Melanoma	N=4	25%	[84]
Basocellular carcinoma of the skin	N=49	2%	[88]
CNS carcinoma	N=55	4%	[77, 88]
Leukemia	N=6	17%	[77]
Uterus cancer	N=49	2%	[88]

*1 An additional case of breast cancer in a biallelic *MUTYH* mutation carrier was reported by Olschwang *et al*. 2007, however no information on sex proportions in the examined MAP patients was reported [88].

*2 This Patient was found to have biallelic *MUTYH* mutations, although no colorectal adenomas were found at the age of 53 years old. However, one case of be early-onset CRC (before 50 years of age) was seen in the patient’s family. This patient had both endometrial cancer as well as sebaceous carcinoma [37].

**Table 5.** *MUTYH* Germline Mutations Among Polyposis Patients who are Negative for *APC* Germline Mutations

	Sieber *et al*. 2003 [40]	Sampson *et al*. 2003 [62]	Isidro *et al*. 2004 [69]	Wang *et al*. 2004 [57]	Nielsen *et al*. 2005 [87]	Russell *et al*. 2006 [75]	Slová *et al*. 2007 [76]	Kim *et al*. 2007 [61]
Population group	UK	UK	Portugal	USA	The Netherlands	Switzerland	Czech Republic	Korea
Number of non-related tested Polyposis patients*1	N=259 (3 to >100)	N =111 (At least 10)	N =53 (10 to >1000)	N=140 (4 to >500)	N=170	N=61 (5-99)	N= 82 (3 to >100)	N=62 (10 to >100)
Method of screening of the *MUTYH* gene	All Exons and Exon-intron boundries	Caucasians:Exon 7 +13*2, Heterozygote/Non-Caucasians: All exons	All Exons	Exon 7 + 13	All Exons	Exon 7 + 13, if hetero-zygote: all exons	All Exons and Exon-intron boundries	All exons
Carriers of monoallelic *MUTYH* germline mutations	4%	3%	0%	3%	24%	10%	1%	5%
Carriers of biallelic *MUTYH*germline mutations	5%	23%	40%	11%	24%	10%	2%	3%

In this table only studies in which the numbers of tested individuals are > 50 are included. The tested individuals are all *APC* germline mutation negative. The individuals tested are either probands or call-up patients with colorectal adenomas.

*1 The groups of tested Polyposis patients included both patients with < and > 100 colorectal adenomas. The reported number of adenomas in the groups in question are given in brackets.

*2 The common mutations Y179C and G396D are found in exon 7 and 13, respectively.

## ABBREVIATIONS

A: = Adenine
AFAP: = Attenuated Familial Adenomatous Polyposis
APC: = Adenomatous Polyposis Coli
BER: = Base Excision Repair
C: = Cytosine
CRC: = Colorectal Cancer
FAP: = Familial Adenomatous Polyposis
G: = Guanine
HhH: = Helix-Hairpin-Helix
HNPCC: = Hereditary Non-Polyposis Colorectal Cancer
LOH: = Loss of Heterozygosity
LOVD: = Leiden Open Variation Database
MAP: = *MUTYH* Associated Polyposis
MMR: = Mismatch Repair
MSI: = Microsatellite Instability
MUTYH gene: = MYH gene = MutY Human Homolog gene
ROS: = Reactive Oxygen Species
8-oxoG: = 8-oxo-7,8-dihydroxy-2´-deoxyguano-sine

## References

[1] Parkin DM, Bray F, Ferlay J, Pisani P (2005). Global cancer statistics, 2002. CA: A cancer journal for clinicians.

[2] Lichtenstein P, Holm NV, Verkasalo PK, Iliadou A, Kaprio J, Koskenvuo M, Pukkala E, Skytthe A, Hemminki K (2000). Environmental and heritable factors in the causation of cancer-analyses of cohorts of twins from Sweden, Denmark, and Finland. N. Engl. J. Med.

[3] Vasen HF, Watson P, Mecklin JP, Lynch HT (1999). New clinical criteria for hereditary nonpolyposis colorectal cancer (HNPCC, Lynch syndrome) proposed by the International Collaborative group on HNPCC. Gastroenterology.

[4] Umar A, Boland CR, Terdiman JP, Syngal S, de la Chapelle A, Ruschoff J, Fishel R, Lindor NM, Burgart LJ, Hamelin R, Hamilton SR, Hiatt RA, Jass J, Lindblom A, Lynch HT, Peltomaki P, Ramsey SD, Rodriguez-Bigas MA, Vasen HF, Hawk ET, Barrett JC, Freedman AN, Srivastava S (2004). Revised Bethesda Guidelines for hereditary nonpolyposis colorectal cancer (Lynch syndrome) and microsatellite instability. J. Natl. Cancer Inst.

[5] Lynch HT, Lynch J (2000). Lynch syndrome: genetics, natural history, genetic counseling, and prevention. J. Clin. Oncol.

[6] Vasen HF (2005). Clinical description of the Lynch syndrome [hereditary nonpolyposis colorectal cancer (HNPCC)]. Fam. Cancer.

[7] Abdel-Rahman WM, Mecklin JP, Peltomaki P (2006). The genetics of HNPCC: application to diagnosis and screening. Crit. Rev. Oncol. Hematol.

[8] Abdel-Rahman WM, Peltomaki P (2004). Molecular basis and diagnostics of hereditary colorectal cancers. Ann. Med.

[9] Vasen HF, Moslein G, Alonso A, Bernstein I, Bertario L, Blanco I, Burn J, Capella G, Engel C, Frayling I, Friedl W, Hes FJ, Hodgson S, Mecklin J-P, Møller P, Nagengast F, Parc Y, Renkonen-Sinisalo L, Sampson JR, Stormorken A, Wijnen J (2007). Guidelines for the clinical management of Lynch syndrome (hereditary non-polyposis cancer). J. Med. Genet.

[10] Bulow S (2003). Results of national registration of familial adenomatous polyposis. Gut.

[11] Fearnhead NS, Britton MP, Bodmer WF (2001). The ABC of APC. Hum. Mol. Genet.

[12] Näthke I (2004). APC at a glance. J. Cell Sci.

[13] Galiatsatos P, Foulkes WD (2006). Familial adenomatous polyposis. Am. J. Gastroenterol.

[14] Lipton L, Tomlinson I (2006). The genetics of FAP and FAP-like syndroms. Fam. Cancer.

[15] Varesco L (2004). Familial adenomatous polyposis: genetics and epidemiology. Tech. Coloproctol.

[16] Vasen HF, Moeslein G, Alonso A, Aretz S, Bernstein I, Bertario L, Blanco I, Bulow S, Burn J, Capella G, Colas C, Engel C, Frayling I, Friedl W, Hes F, Hodgson S, Jarvinen H, Mecklin J-P, Møller P, Myrhøj T, Nagengast FM, Parc Y, Phillips R, Clark S, Ponz de Leon M, Renkonen-Sinisalo L, Sampson J, Stormorken A, Tejpar S, Thomas H, Wijnen J (2008). Guidelines for the clinical management of familial adenomatous polyposis (FAP). Gut.

[17] Nieuwenhuis MH, Vasen HF (2007). Correlations between mutation site in APC and phenotype of familial adenomatous polyposis (FAP): a review of the literature. Crit. Rev. Oncol. Hematol.

[18] Lamlum H, Al Tassan N, Jaeger E, Frayling I, Sieber O, Reza FB, Eckert M, Rowan A, Barclay E, Atkin W, Williams C, Gilbert J, Cheadle J, Bell J, Houlston R, Bodmer W, Sampson J, Tomlinson I (2000). Germline APC variants in patients with multiple colorectal adenomas, with evidence for the particular importance of E1317Q. Hum. Mol. Genet.

[19] Nielsen M, Hes FJ, Nagengast FM, Weiss MM, Mathus-Vliegen EM, Morreau H, Breuning MH, Wijnen JT, Tops CM, Vasen HF (2007). Germline mutations in APC and MUTYH are responsible for the majority of families with attenuated familial adenomatous polyposis. Clin. Genet.

[20] Mecklin JP, Jarvinen HJ (2005). Surveillance in Lynch Syndrom. Fam. Cancer.

[21] Al-Tassan N, Chmiel NH, Maynard J, Fleming N, Livingston AL, Williams GT, Hodges AK, Davies DR, David SS, Sampson JR, Cheadle JP (2002). Inherited variants of MYH associated with somatic G:C-->T:A mutations in colorectal tumors. Nat. Genet.

[22] Cheadle JP, Sampson JR (2007). MUTYH-associated polyposis -from defect in base excision repair to clinical genetic testing. DNA Repair.

[23] Cheadle JP, Sampson JR (2003). Exposing the MYtH about base excision repair and human inherited disease. Hum. Mol. Genet.

[24] Sampson JR, Jones S, Dolwani S, Cheadle JP (2005). MutYH (MYH) and colorectal cancer. Biochem. Soc. Trans.

[25] Nohmi T, Kim SR, Yamada M (2005). Modulation of oxidative mutagenesis and carcinogenesis by polymorphic forms of human DNA repair enzymes. Mutat. Res.

[26] David SS, O'Shea VL, Kundu S (2007). Base-excision repair of oxidative DNA damage. Nature.

[27] Chow E, Thirlwell C, Macrae F, Lipton L (2004). Colorectal cancer and inherited mutations in base-excision repair. Lancet Oncol.

[28] Lipton L, Tomlinson I (2004). The multiple colorectal adenoma phenotype and MYH, a base excision repair gene. Clin. Gastroenterol. Hepatol.

[29] Parker AR, Eshleman JR (2003). Human MutY: gene structure, protein functions and interactions, and role in carcinogenesis. Cell Mol. Life Sci.

[30] Guan Y, Manuel RC, Arvai AS, Parikh SS, Mol CD, Miller JH, Lloyd S, Tainer JA (1998). MutY catalytic core, mutant and bound adenine structures define specificity for DNA repair enzyme superfamily. Nat. Struct. Biol.

[31] Lu AL, Bai H, Shi G, Chang DY (2006). MutY and MutY homologs (MYH) in genome maintenance. Front. Biosci.

[32] Volk DE, House PG, Thiviyanathan V, Luxon BA, Zhang S, Lloyd RS, Gorenstein DG (2000). Structural similarities between MutT and the C-terminal domain of MutY. Biochemistry.

[33] Shinmura K, Yamaguchi S, Saitoh T, Kohno T, Yokota J (2001). Somatic mutations and single nucleotide polymorphisms of base excision repair genes involved in the repair of 8-hydroxyguanine in damaged DNA. Cancer lett.

[34] Tao H, Shinmura K, Hanaoka T, Natsukawa S, Shaura K, Koizumi Y, Kasuga Y, Ozawa T, Tsujinaka T, Li Z, Yamaguchi S, Yokota J, Sugimura H, Tsugane S (2004). A novel splice-site variant of the base excision repair gene MYH is associated with production of an aberrant mRNA transcript encoding a truncated MYH protein not localized in the nucleus. Carcinogenesis.

[35] Kim CJ, Cho YG, Park CH, Kim SY, Nam SW, Lee SH, Yoo NJ, Lee JY, Park WS (2004). Genetic alterations of the MYH gene in gastric cancer. Oncogene.

[36] Yamaguchi S, Shinmura K, Saitoh T, Takenoshita S, Kuwano H, Yokota J (2002). A single nucleotide polymorphism at the splice donor site of the human MYH base excision repair genes results in reduced translation efficiency of its transcripts. Genes Cells.

[37] Barnetson RA, Devlin L, Miller J, Farrington SM, Slater S, Drake AC, Campbell H, Dunlop MG, Porteous ME (2007). Germline mutation prevalence in the base excision repair gene, MYH, in patients with endometrial cancer. Clin. Genet.

[38] Jones S, Emmerson P, Maynard J, Best JM, Jordan S, Williams GT, Sampson JR, Cheadle JP (2002). Biallelic germline mutations in MYH predispose to multiple colorectal adenoma and somatic G:C-->T:A mutations. Hum. Mol. Genet.

[39] Halford SE, Rowan AJ, Lipton L, Sieber OM, Pack K, Thomas HJ, Hodgson SV, Bodmer WF, Tomlinson IP (2003). Germline mutations but not somatic changes at the MYH locus contribute to the pathogenesis of unselected colorectal cancers. Am. J. Pathol.

[40] Sieber OM, Lipton L, Crabtree M, Heinimann K, Fidalgo P, Phillips RK, Bisgaard ML, Orntoft TF, Aaltonen LA, Hodgson SV, Thomas HJW, Tomlinson I (2003). Multiple colorectal adenomas, classic adenomatous polyposis, and germ-line mutations in MYH. N. Engl. J. Med.

[41] Kury S, Buecher B, Robiou-du-Pont S, Scoul C, Colman H, Lelievre B, Olschwang S, Le Houerou C, Le Neel T, Faroux R, Ollivry J, Lafraise B, Chupin L-D, Bézieau S (2007). The Thorough Screening of the MUTYH Gene in a Large French Cohort of Sporadic Colorectal Cancers. Genetic Testing.

[42] Kim JC, Ka IH, Lee YM, Koo KH, Kim HC, Yu CS, Jang SJ, Kim YS, Lee HI, and Lee KH (2007). MYH, OGG1, MTH1, and APC alterations involved in the colorectal tumorigenesis of Korean patients with multiple adenomas. Virchows Arch.

[43] Kim IJ, Ku JL, Kang HC, Park JH, Yoon KA, Shin Y, Park HW, Jang SG, Lim SK, Han SY, Shin Y-K, Lee MR, Jeong S-Y, Shin H-R, Lee JS, Kim W-H, Park J-G (2004). Mutational analysis of OGG1, MYH, MTH1 in FAP, HNPCC and sporadic colorectal cancer patients: R154H OGG1 polymorphism is associated with sporadic colorectal cancer patients. Hum. Genet.

[44] Fokkema IF, den Dunnen JT, Taschner PE (2005). LOVD: easy creation of a locus-specific sequence variation database using an “LSDB-in-a-box” approach. Hum. Mutat.

[45] Out A, Hes F, Tops C Personal communication on 13.03.2008..

[46] Lipton L, Halford SE, Johnson V, Novelli MR, Jones A, Cummings C, Barclay E, Sieber O, Sadat A, Bisgaard ML, Hodgson SV, Aaltonen LA, Thomas HJ, Tomlinson IP (2003). Carcinogenesis in MYH-associated polyposis follows a distinct genetic pathway. Cancer Res.

[47] Jones S, Lambert S, Williams GT, Best JM, Sampson JR, Cheadle JP (2004). Increased frequency of the k-ras G12C mutation in MYH polyposis colorectal adenomas. Br. J. Cancer.

[48] Kambara T, Whitehall VL, Spring KJ, Barker MA, Arnold S, Wynter CV, Matsubara N, Tanaka N, Young JP, Leggett BA, Jass JR (2004). Role of inherited defects of MYH in the development of sporadic colorectal cancer. Genes Chromosomes Cancer.

[49] Chmiel NH, Livingston AL, David SS (2003). Insight into the functional consequences of inherited variants of the hMYH adenine glycosylase associated with colorectal cancer: complementation assays with hMYH variants and pre-steady-state kinetics of the corresponding mutated *E. coli* enzymes. J. Mol. Biol.

[50] Johnson V, Lipton LR, Cummings C, Eftekhar Sadat AT, Izatt L, Hodgson SV, Talbot IC, Thomas HJ, Silver AJ, Tomlinson IP (2005). Analysis of somatic molecular changes, clinicopathological features, family history, and germline mutations in colorectal cancer families: evidence for efficient diagnosis of HNPCC and for the existence of distinct groups of non-HNPCC families. J. Med. Genet.

[51] Bougatef K, Marrakchi R, Kourda N, Ben Lahely YB, Jileni SB, El Khil HK, Soubrier F, Ben Ammar Elgaaied A (2007). Somatic mutation of MutYH in Tunisian patients with sporadic colorectal cancer. J. Clin. Lab. Anal.

[52] Edelmann L, Edelmann W (2004). Loss of DNA mismatch repair function and cancer predisposition in the mouse: animal models for human hereditary nonpolyposis colorectal cancer. Am. J. Med. Genet.

[53] Gu Y, Parker A, Wilson TM, Bai H, Chang DY, Lu AL (2002). Human MutY homolog, a DNA glycosylase involved in base excision repair, physically and functionally interacts with mismatch repair proteins human MutS homolog 2/human MutS homolog 6. J. Biol. Chem.

[54] Bai H, Jones S, Guan X, Wilson TM, Sampson JR, Cheadle JP, Lu AL (2005). Functional characterization of two human MutY homolog (hMYH) missense mutations (R227W and V232F) that lie within the putative hMSH6 binding domain and are associated with hMYH polyposis. Nucleic Acids Res.

[55] Miyaki M, Iijima T, Yamaguchi T, Hishima T, Tamura K, Utsunomiya J, Mori T (2005). Germline mutations of the MYH gene in Japanese patients with multiple colorectal adenomas. Mutat. Res.

[56] Niessen RC, Sijmons RH, Ou J, Olthof SG, Osinga J, Ligtenberg MJ, Hogervorst FB, Weiss MM, Tops CM, Hes FJ, de Bock GH, Buys CH, Kleibeuker JH, Hofstra RM (2006). MUTYH and the mismatch repair system: partners in crime?. Hum. Genet.

[57] Wang L, Baudhuin LM, Boardman LA, Steenblock KJ, Petersen GM, Halling KC, French AJ, Johnson RA, Burgart LJ, Rabe K, Lindor NM, Thibodeau SN (2004). MYH mutations in patients with attenuated and classic polyposis and with young-onset colorectal cancer without polyps. Gastroenterology.

[58] van Puijenbroek M, Nielsen M, Reinards TH, Weiss MM, Wagner A, Hendriks YM, Vasen HF, Tops CM, Wijnen J, van Wezel T, Hes FJ, Morreau H (2007). The natural history of a combined defect in MSH6 and MUTYH in a HNPCC family. Fam. Cancer.

[59] Cao X, Hong Y, Eu KW, Loi C, Cheah PY (2006). Singapore familial adenomatous polyposis (FAP) patients with classical adenomatous polyposis but undetectable APC mutations have accelerated cancer progression. Am. J. Gastroenterol.

[60] Kim H, Kim HJ, Chi SG, Lee SK, Joo GR, Dong SH, Kim BH, Chang YW, Lee JI, Chang R (2006). Absence of MutY homologue mutation in patients with multiple sporadic adenomatous polyps in Korea. World J. Gastroenterol.

[61] Kim DW, Kim IJ, Kang HC, Jang SG, Kim K, Yoon HJ, Ahn SA, Han SY, Hong SH, Hwang JA, Sohn DK, Jeong S-H, Choi HS, Hong CW, Lim S-B, Park J-G (2007). Germline mutations of the MYH gene in Korean patients with multiple colorectal adenomas. Int. J. Colorectal Dis.

[62] Sampson JR, Dolwani S, Jones S, Eccles D, Ellis A, Evans DG, Frayling I, Jordan S, Maher ER, Mak T, Maynard J, Pigatto F, Shaw J, Cheadle JP (2003). Autosomal recessive colorectal adenomatous polyposis due to inherited mutations of MYH. Lancet.

[63] Ajith Kumar VK, Gold JA, Mallon E, Thomas S, Hodgson SV (2007). Sebaceous adenomas in an MYH associated polyposis patient of Indian (Gujarati) origin. Fam. Cancer.

[64] Dolwani S, Williams GT, West KP, Newman J, Stock D, Griffiths AP, Best J, Cheadle JP, Sampson JR (2007). Analysis of inherited MYH/(MutYH) mutations in British Asian patients with colorectal cancer. Gut.

[65] Venesio T, Molatore S, Cattaneo F, Arrigoni A, Risio M, Ranzani GN (2004). High frequency of MYH gene mutations in a subset of patients with familial adenomatous polyposis. Gastroenterology.

[66] Aceto G, Cristina Curia M, Veschi S, De Lellis L, Mammarella S, Catalano T, Stuppia L, Palka G, Valanzano R, Tonelli F, Casale V, Stigliano V, Cetta F, Battista P, Mariani-Costantini R, Cama A (2005). Mutations of APC and MYH in unrelated Italian patients with adenomatous polyposis coli. Hum. Mutat.

[67] Alhopuro P, Parker AR, Lehtonen R, Enholm S, Jarvinen HJ, Mecklin JP, Karhu A, Eshleman JR, Aaltonen LA (2005). A novel functionally deficient MYH variant in individuals with colorectal adenomatous polyposis. Hum. Mutat.

[68] Leite JS, Isidro G, Martins M, Regateiro F, Albuquerque O, Amaro P, Romaozinho JM, Boavida G, Castro-Sousa F (2005). Is prophylactic colectomy indicated in patients with MYH-associated polyposis?. Colorectal Dis.

[69] Isidro G, Laranjeira F, Pires A, Leite J, Regateiro F, Castro e Sousa F, Soares J, Castro C, Giria J, Brito MJ, Medaira A, Teixeira R, Morna H, Gaspar I, Marinho C, Jorge R, Brehm A, Ramos JS, Boavida MG (2004). Germline MUTYH (MYH) mutations in Portuguese individuals with multiple colorectal adenomas. Hum. Mutat.

[70] Croitoru ME, Cleary SP, Di Nicola N, Manno M, Selander T, Aronson M, Redston M, Cotterchio M, Knight J, Gryfe R, Gallinger S (2004). Association between biallelic and monoallelic germline MYH gene mutations and colorectal cancer risk. J. Natl. Cancer Inst.

[71] Fleischmann C, Peto J, Cheadle J, Shah B, Sampson J, Houlston RS (2004). Comprehensive analysis of the contribution of germline MYH variation to early-onset colorectal cancer. Int. J. Cancer.

[72] Kairupan CF, Meldrum CJ, Crooks R, Milward EA, Spigel-man AD, Burgess B, Groombridge C, Kirk J, Tucker K, Ward R, Williams R, Scott RJ (2005). Mutation analysis of the MYH gene in an Australian series of colorectal polyposis patients with or without germline APC mutations. Int. J. Cancer.

[73] Zhou XL, Djureinovic T, Werelius B, Lindmark G, Sun XF, Lindblom A (2005). Germline mutations in the MYH gene in Swedish familial and sporadic colorectal cancer. Genetic Testing.

[74] Kanter-Smoler G, Bjork J, Fritzell K, Engwall Y, Hallberg B, Karlsson G, Gronberg H, Karlsson P, Wallgren A, Wahlstrom J, Hultcrantz R, Nordling M (2006). Novel findings in Swedish patients with MYH-associated polyposis: mutation detection and clinical characterization. Clin. Gastroenterol. Hepatol.

[75] Russell AM, Zhang J, Luz J, Hutter P, Chappuis PO, Berthod CR, Maillet P, Mueller H, Heinimann K (2006). Prevalence of MYH germline mutations in Swiss APC mutation-negative polyposis patients. Int. J. Cancer.

[76] Sulova M, Zidkova K, Kleibl Z, Stekrova J, Kebrdlova V, Bortlik M, Lukas M, Kohoutova M (2007). Mutation analysis of the MYH gene in unrelated Czech APC mutation-negative polyposis patients. Eur. J. Cancer.

[77] Croitoru ME, Cleary SP, Berk T, Di Nicola N, Kopolovic I, Bapat B, Gallinger S (2007). Germline MYH mutations in a clinic-based series of Canadian multiple colorectal adenoma patients. J. Surg. Oncol.

[78] Lejeune S, Guillemot F, Triboulet JP, Cattan S, Mouton C, Porchet N, Manouvrier S, Buisine MP (2006). Low frequency of AXIN2 mutations and high frequency of MUTYH mutations in patients with multiple polyposis. Hum. Mutat.

[79] Fromme JC, Banerjee A, Huang SJ, Verdine GL (2004). Structural basis for removal of adenine mispaired with 8-oxoguanine by MutY adenine DNA glycosylase. Nature.

[80] Chmiel NH, Golinelli MP, Francis AW, David SS (2001). Efficient recognition of substrates and substrate analogs by the adenine glycosylase MutY requires the C-terminal domain. Nucleic Acids Res.

[81] Pope MA, Chmiel NH, David SS (2005). Insight into the functional consequences of hMYH variants associated with colorectal cancer: distinct differences in the adenine glycosylase activity and the response to AP endonucleases of Y150C and G365D murine MYH. DNA Repair.

[82] Ma H, Lee HM, Englander EW (2004). N-terminus of the rat adenine glycosylase MYH affects excision rates and processing of MYH-generated abasic sites. Nucleic Acids Res.

[83] Parker AR, Sieber OM, Shi C, Hua L, Takao M, Tomlinson IP, Eshleman JR (2005). Cells with pathogenic biallelic mutations in the human MUTYH gene are defective in DNA damage binding and repair. Carcinogenesis.

[84] Enholm S, Hienonen T, Suomalainen A, Lipton L, Tomlinson I, Karja V, Eskelinen M, Mecklin JP, Karhu A, Jarvinen HJ, Aaltonen LA (2003). Proportion and phenotype of MYH-associated colorectal neoplasia in a population-based series of Finnish colorectal cancer patients. Am. J. Pathol.

[85] Aretz S, Uhlhaas S, Goergens H, Siberg K, Vogel M, Pagenstecher C, Mangold E, Caspari R, Propping P, Friedl W (2006). MUTYH-associated polyposis: 70 of 71 patients with biallelic mutations present with an attenuated or atypical phenotype. Int. J. Cancer.

[86] Bouguen G, Manfredi S, Blayau M, Dugast C, Buecher B, Bonneau D, Siproudhis L, David V, Bretagne JF (2007). Colorectal adenomatous polyposis Associated with MYH mutations: genotype and phenotype characteristics. Dis. Colon Rectum.

[87] Nielsen M, Franken PF, Reinards TH, Weiss MM, Wagner A, van der Klift H, Kloosterman S, Houwing-Duistermaat JJ, Aalfs CM, Ausems MG, Bröcker-Vriends AH, Gomez Garcia MH, Hoogerbrugge N, Menko FH, Sijmons RH, Verhoef S, Kuipers EJ, Morreau H, Breuning MH, Tops CM, Wijnen JT, Vasen HF, Fodde R, Hes FJ (2005). Multiplicity in polyp count and extracolonic manifestations in 40 Dutch patients with MYH associated polyposis coli (MAP). J. Med. Genet.

[88] Olschwang S, Blanche H, de Moncuit C, Thomas G (2007). Similar colorectal cancer risk in patients with monoallelic and biallelic mutations in the MYH gene identified in a population with adenomatous polyposis. Genetic Testing.

[89] Jenkins MA, Croitoru ME, Monga N, Cleary SP, Cotterchio M, Hopper JL, Gallinger S (2006). Risk of colorectal cancer in monoallelic and biallelic carriers of MYH mutations: a population-based case-family study. Cancer Epidemiol. Biomarkers Prev.

[90] Peterlongo P, Mitra N, Sanchez de Abajo A, de la Hoya M, Bassi C, Bertario L, Radice P, Glogowski E, Nafa K, Caldes T, Offit K, Ellis NA (2006). Increased frequency of disease-causing MYH mutations in colon cancer families. Carcinogenesis.

[91] Peterlongo P, Mitra N, Chuai S, Kirchhoff T, Palmer C, Huang H, Nafa K, Offit K, Ellis NA (2005). Colorectal cancer risk in individuals with biallelic or monoallelic mutations of MYH. Int. J. Cancer.

[92] Farrington SM, Tenesa A, Barnetson R, Wiltshire A, Pren-dergast J, Porteous M, Campbell H, Dunlop MG (2005). Germline susceptibility to colorectal cancer due to base-excision repair gene defects. Am. J. Hum. Genet.

[93] Gismondi V, Meta M, Bonelli L, Radice P, Sala P, Bertario L, Viel A, Fornasarig M, Arrigoni A, Gentile M, Ponz De Leon M, Anselmi L, Mareni C, Bruzzi P, Varesco L (2004). Prevalence of the Y165C, G382D and 1395delGGA germline mutations of the MYH gene in Italian patients with adenomatous polyposis coli and colorectal adenomas. Int. J. Cancer.

[94] Nielsen M, Poley JW, Verhoef S, van Puijenbroek M, Weiss MM, Burger GT, Dommering CJ, Vasen HF, Kuipers EJ, Wagner A, Morreau H, Hes FJ (2006). Duodenal carcinoma in MUTYH-associated polyposis. J. Clin. Pathol.

[95] Ponti G, Ponz de Leon M, Maffei S, Pedroni M, Losi L, Di Gregorio C, Gismondi V, Scarselli A, Benatti P, Roncari B, Seidenari S, Pellacani G, Varotti C, Prete E, Varesco L, Roncucci L (2005). Attenuated familial adenomatous polyposis and Muir-Torre syndrome linked to compound biallelic constitutional MYH gene mutations. Clin. Genet.

[96] Baglioni S, Melean G, Gensini F, Santucci M, Scatizzi M, Papi L, Genuardi M (2005). A kindred with MYH-associated polyposis and pilomatricomas. Am. J. Med. Genet. A.

[97] Tenesa A, Campbell H, Barnetson R, Porteous M, Dunlop M, Farrington SM (2006). Association of MUTYH and colorectal cancer. Br. J. Cancer.

[98] Webb EL, Rudd MF, Houlston RS (2006). Colorectal cancer risk in monoallelic carriers of MYH variants. Am. J. Hum. Genet.

[99] Tenesa A, Farrington SM, Dunlop MG (2005). Re: Association between biallelic and monoallelic germline MYH gene mutations and colorectal cancer risk. J. Natl. Cancer Inst.

[100] Eliason K, Hendrickson BC, Judkins T, Norton M, Leclair B, Lyon E, Ward B, Noll W, Scholl T (2005). The potential for increased clinical sensitivity in genetic testing for polyposis colorectal cancer through the analysis of MYH mutations in North American patients. J. Med. Genet.

[101] Piccioli P, Serra M, Gismondi V, Pedemonte S, Loiacono F, Lastraioli S, Bertario L, De Angioletti M, Varesco L, Notaro R (2006). Multiplex tetra-primer amplification refractory mutation system PCR to detect 6 common germline mutations of the MUTYH gene associated with polyposis and colorectal cancer. Clin. Chem.

[102] van Puijenbroek M, Nielsen M, Tops CM, Halfwerk H, Vasen HF, Weiss MM, van Wezel T, Hes FJ, Morreau H (2008). Identification of Patients with (Atypical) MUTYH-Associated Polyposis by KRAS2 c.34G > T Prescreening Followed by MUTYH Hotspot Analysis in Formalin-Fixed Paraffin-Embedded Tissue. Clin. Cancer Res.

